# Electroacupuncture-Induced Plasticity between Different Representations in Human Motor Cortex

**DOI:** 10.1155/2020/8856868

**Published:** 2020-08-14

**Authors:** Weiqin Peng, Tiange Yang, Jiawei Yuan, Jianpeng Huang, Jianhua Liu

**Affiliations:** Acupuncture Research Team, the Second Affiliated Hospital of Guangzhou University of Chinese Medicine, Guangzhou, China

## Abstract

Somatosensory stimulation can effectively induce plasticity in the motor cortex representation of the stimulated body part. Specific interactions have been reported between different representations within the primary motor cortex. However, studies evaluating somatosensory stimulation-induced plasticity between different representations within the primary motor cortex are sparse. The purpose of this study was to investigate the effect of somatosensory stimulation on the modulation of plasticity between different representations within the primary motor cortex. Twelve healthy volunteers received both electroacupuncture (EA) and sham EA at the TE5 acupoint (located on the forearm). Plasticity changes in different representations, including the map volume, map area, and centre of gravity (COG) were evaluated by transcranial magnetic stimulation (TMS) before and after the intervention. EA significantly increased the map volume of the forearm and hand representations compared to those of sham EA and significantly reduced the map volume of the face representation compared to that before EA. No significant change was found in the map volume of the upper arm and leg representations after EA, and likewise, no significant changes in map area and COG were observed. These results suggest that EA functions as a form of somatosensory stimulation to effectively induce plasticity between different representations within the primary motor cortex, which may be related to the extensive horizontal intrinsic connectivity between different representations. The cortical plasticity induced by somatosensory stimulation might be purposefully used to modulate human cortical function.

## 1. Introduction

In the mid-20th century, Penfield and colleagues described a somatotopic map of the human primary motor cortex, which revealed the rough distribution of body part representations in the human cortex [1]. The cortical representations of adjacent body parts have been shown to extensively overlap [2], and furthermore, the motor cortex reportedly contains a network of intracortical fibres that interconnect various cortical motor representations [3]. This network is dynamic and contains an anatomical substrate for topographic plasticity.

Somatosensory stimulation has been proved to modulate plasticity in the primary motor cortex [4], which is mainly defined as a change in the excitability and area of a cortical representation. In healthy subjects, short-term electrical stimulation over a peripheral nerve or a muscle motor point can increase the motor cortical excitability of the stimulated body parts [5]. Alternatively, the application of long-term daily transcutaneous nerve electrical stimulation (TENS) to a hand muscle has been shown to result in an enlargement in the cortical representation of the stimulated muscle [6]. Acupuncture, a safe, painless, and easily performed method of somatosensory stimulation, can also modulate plasticity in the primary motor cortex [7]. This has been demonstrated, for example, by several functional magnetic resonance imaging (fMRI) studies showing that acupuncture at the GB34 acupoint of the leg activates the motor cortex in healthy subjects [8] and in patients with Parkinson's disease [9]. Moreover, a transcranial magnetic stimulation (TMS) study showed that acupuncture at LI4 of the hand significantly changes the motor evoked potentials (MEP) amplitude of hand muscle in healthy subjects [10].

However, much less is known about somatosensory stimulation-induced plasticity between different representations within the motor cortex. There is evidence that noninvasive brain stimulation of motor cortex might induce across-representation plasticity in the primary motor cortex. Repetitive transcranial magnetic stimulation (rTMS) of the face or hand representations leads to an inhibition activity of the adjacent arm representation during transient ischemic nerve block in healthy subjects [11]. Additionally, a number of studies have shown that plasticity occurs between different representations in motor cortex in cases of peripheral nerve lesion [12, 13], brain injury [14], motor practice [15], and motor learning [16]. More importantly, modulation of the plasticity between different representations has been proposed to play a key role in functional recovery in animals with lesions [17, 18] and in patients with neurological disorders [19–21]. To date, although somatosensory stimulation treatments, including electroacupuncture (EA), have been widely used for the rehabilitation of neurological function [22–24], their mechanisms of action are still unknown.

The present study used EA to apply somatosensory stimulation to the forearm and investigated the changes in the cortical representations of the forearm, hand, upper arm, face, and leg in healthy volunteers. Changes in motor cortex representations were evaluated using a TMS protocol to test differences in the excitability and size of the cortical representation [25]. Based on previous findings that central stimulation induces plasticity within the primary motor cortex [11], we hypothesized that EA of the forearm would induce a global effect on different representations, which might provide a new approach for the treatment of neurological diseases.

## 2. Materials and Methods

### 2.1. Subjects and Experimental Design

Twelve healthy, right-handed adults (aged 26–35 years; 1 male) participated in the study. The subjects had not undergone acupuncture in the month before beginning the study and none had neurological, psychiatric, or any other medical problems or had reported any contraindications to TMS [26]. All participants were informed about the potential benefits and risks of the study and provided written consent to participate. The present study was approved by the Ethics Committee of the Guangdong Provincial Hospital of Traditional Chinese Medicine (approval no.BF2019-040-01).

**Figure 1:**
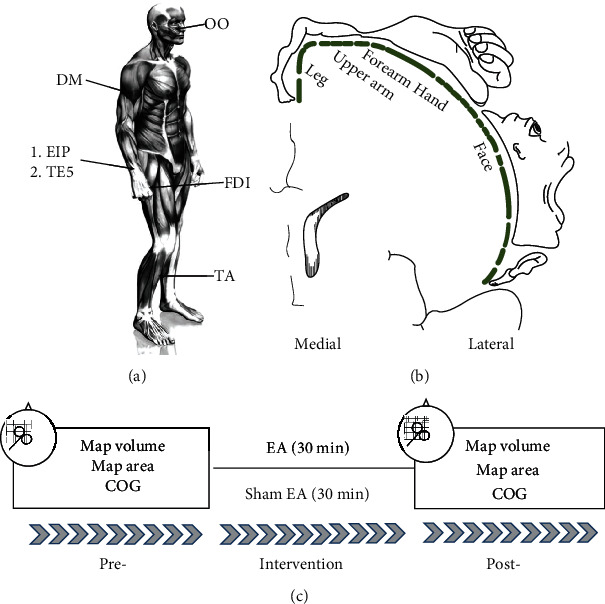
(a) Schematic drawings of a subject, showing the location of acupoint TE5 (Waiguan) and the target muscles. (b) Penfield and Rasmussen's homunculus, in which representations of the leg, upper arm, forearm, hand, and face muscles are roughly arranged in a medial-to-lateral direction. (c) Experimental paradigm. All subjects participated in EA and sham EA groups. Abbreviations: COG: centre of gravity; DM: deltoid muscle; EA: electroacupuncture; EIP: extensor indicis proprius; FDI: first dorsal interosseous; OO: orbicularis oculi; TA: tibialis anterior; TE5: Waiguan acupoint.

The study timeline is depicted in Figure 1(c). Each subject received real and sham EA in separate sessions, with a washout period of at least 2 weeks. The order of interventions (real–sham or sham–real) was chosen randomly for each participant. The study is registered in the Chinese Clinical Trial Registry (no. ChiCTR-1900026290).

### 2.2. Intervention

The subjects sat comfortably on an armchair and were instructed to stay relaxed during the intervention. Acupuncture was performed by the same experienced acupuncturist under aseptic conditions using disposable acupuncture needles (diameter, 0.25 mm; length, 25 mm; Hwato, Suzhou Medical Appliance Factory, Suzhou, China).

This study used the “Waiguan” (TE5) acupoint, located on the posterior aspect of the forearm, at the midpoint of the interosseous space between the radius and the ulna, 2 B-cun proximal to the dorsal wrist crease [27] (Figure 1(a)). In each EA session, acupuncture was performed at TE5 on the right forearm with a depth of real acupuncture insertion of about 10–15 mm. Upon the subject reporting a De Qi sensation during acupuncture, a nerve stimulator (HANS-200A, Jisheng Medical Technology Limited Company, Nanjing, China) was used to perform EA (2 Hz) at a comfortable intensity depending on the tolerance of the individual. In the sham EA sessions, a noninvasive blunt needle (diameter, 0.25 mm; length, 25 mm) supported by a sponge cushion served as the sham acupuncture setup [28]. The EA and sham EA interventions each lasted for 30 min.

### 2.3. Electromyography (EMG)

EMG records were obtained from the following muscles ipsilateral to the acupuncture sites in the EA sessions: first dorsal interosseous (FDI); extensor indicis proprius (EIP); deltoid muscle (DM); orbicularis oculi (OO); and tibialis anterior (TA). However, only the EIP and FDI were recorded in the sham EA sessions.

Surface electromyography as recorded from the target muscles using 9-mm-diameter Ag/AgCl surface electrodes on a belly–tendon montage. Responses were input into an amplifier through filters with a bandpass of 20–2,000 kHz, then digitized and stored on a computer for later offline analyses. Since corticospinal excitability depends on limb posture [29], all participants maintained a specific limb position, as described below, while being seated comfortably on a chair.

For FDI and EIP recordings, the subjects maintained a static wrist extension and relaxed their fingers with the forearm resting on an armrest. For OO recordings, the subjects were asked to blink slightly to moderately activate the OO muscle. For DM recordings, to activate the middle deltoid muscle in subjects, the middle portions of the deltoids were abducted from the shoulders at a 30° angle, drawing them away from the trunk. For TA recordings, the subjects were constrained by a flexible weight placed over the dorsum of the right foot allowing for 10% of the maximum voluntary isometric contraction.

### 2.4. TMS Protocol

Based on the methods described by Nicolini et al. [25], TMS was applied to the motor cortex in the left hemisphere before and after the intervention (EA/sham EA) with a Magstim Super Rapid magnetic stimulator (Magstim Company, Dyfed, UK) equipped with a figure-of-eight coil (external wing diameter, 70 mm). The coil was orientated at 45°oblique to the sagittal plane so that the induced current flowed in a posterior–anterior direction (Figure 1(c)). All subjects wore a tight-fitting cap with a coordinate (grid, 1 × 1 cm). The resting motor threshold (RMT) was defined as the minimum stimulus intensity that elicited >50 *μ*V peak-to-peak amplitude in 50% of the trials with the muscles (FDI and EIP) at rest (no muscle contraction). In addition, the active motor threshold (AMT) was defined as the minimum stimulus intensity that elicited >100 *μ*V peak-to-peak amplitude in 50% of the trials with muscle contraction (OO, DM, and TA). The motor “hotspot” for each muscle was determined by delivering a single TMS pulse at the motor threshold to each grid point and subsequently identifying the location in the grid with the largest peak-to-peak MEPs. The TMS intensity for mapping was set at 120% of the motor threshold (RMT or AMT). Six successive pulses separated by intervals of 4–5 seconds were delivered to each point of the grid. A grid point was considered responsive if at least three MEPs were elicited. For each muscle, the peak-to-peak MEP amplitudes of three TMS stimuli at each grid point were averaged to determine the maximum average MEP amplitude. Only grid points that elicited an average MEP amplitude ≥20% of the maximum average MEP amplitude were considered active sites and included in the map. The nonactive sites delimited the mapping boundaries.

### 2.5. TMS Outcomes

The extracted TMS variables included the following: (1) map volume, (2) map area, and (3) centre of gravity (COG). The map volume was calculated as the sum of the mean amplitudes at all active sites. A standardized grid (1 × 1 cm) was used across subjects, with the number of active sites accurately representing the map area (cm^2^). The COG was computed using Equations (1) and (2), where *MEPi* represents the mean amplitude of the MEPs produced at one active site [30].

(1) $$\begin{array}{c} COGx=\left({\Sigma x}_{i}\times {MEP}_{i}\right)/{\Sigma MEP}_{i}, \end{array}$$

(2) $$\begin{array}{c} COGy=\left({\Sigma y}_{i}\times {MEP}_{i}\right)/{\Sigma MEP}_{i}. \end{array}$$

### 2.6. Statistical Analysis

The data were analysed using IBM SPSS Statistics for Windows, version 20.0. The data were tested for normality using Shapiro–Wilk normality tests; as some data sets did not meet the normality criteria, nonparametric statistics were used to statistically analyse them. All data are presented as the mean ± standard deviation (SD). Wilcoxon signed-rank tests were used to compare data before (pre-) and after (post-) intervention. Mann–Whitney *U*-tests were used to compare data between groups. *P* < 0.05 was considered statistically significant.

## 3. Results and Discussion

**Figure 2:**
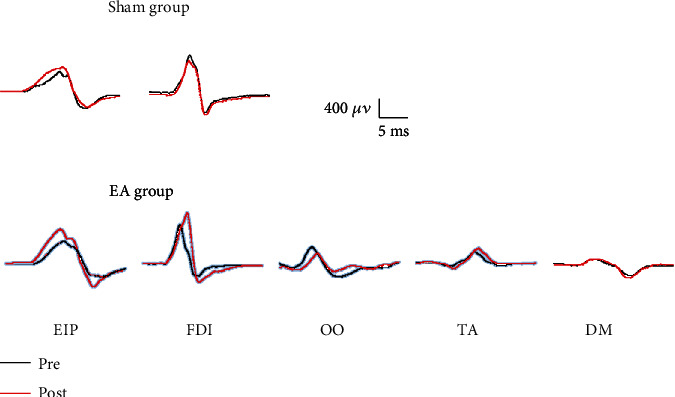
Raw MEP data (single trace) from a representative subject before (black line) and after (red line) each intervention. Abbreviations: DM: deltoid muscle; EA: electroacupuncture; EIP: extensor indicis proprius; FDI: first dorsal interosseous; MEP: motor evoked potential; OO: orbicularis oculi; TA: tibialis anterior.

Twelve participants completed the study procedures without reporting side effects. Raw MEP data from one representative subject is shown in Figure 2.

### 3.1. Effect of EA on Map Volume

**Figure 3:**
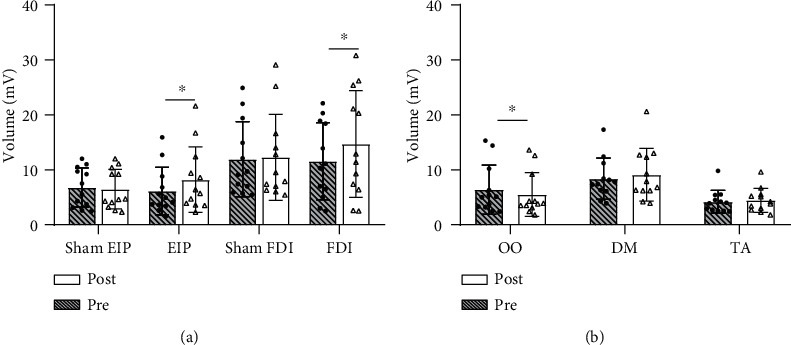
(a, b) Mean (±SD) changes in the map volumes of target muscles plotted with the individual data from each subject before and after the intervention. ^∗^*P* < 0.05, Wilcoxon signed-rank tests. Abbreviations: DM: deltoid muscle; EIP: extensor indicis proprius; FDI: first dorsal interosseous; OO: orbicularis oculi; SD: standard deviation; TA: tibialis anterior.

The effects of EA on map volume are shown in Figure 3. No significant differences between EA and sham EA groups were observed at baseline for the representations of the forearm (EIP; pre-EA, 6.1 ± 4.3 mV vs. presham EA, 6.8 ± 3.6 mV; *P* = 0.603, *Z* = −0.520, Mann–Whitney *U*-tests) and hand (FDI; pre-EA, 11.6 ± 7.0 mV vs. presham EA, 12.0 ± 6.8 mV; *P* = 0.795, *Z* = −0.260, Mann–Whitney *U*-tests). For both of these representations, significant increases were observed in the EA group following the intervention (EIP: pre-EA, 6.1 ± 4.3 mV vs. post-EA, 8.2 ± 5.9 mV, *P* = 0.028, *Z* = −2.197; FDI: pre-EA, 11.6 ± 7.0 mV vs. post-EA, 11.6 ± 7.0 mV, *P* = 0.008, *Z* = −2.667; Wilcoxon signed-rank tests), while no significant change was observed in the sham EA group (EIP: presham EA, 6.8 ± 3.6 mV vs. postsham EA, 6.5 ± 3.6 mV, *P* = 1.000, *Z* = 0.000; FDI: presham EA, 12.0 ± 6.8 mV vs. postsham EA, 12.3 ± 7.8 mV, *P* = 0.937, Z = −0.078; Wilcoxon signed-rank tests). Furthermore, significant between-group effects of EA on the forearm and hand representations were observed (EIP: EA, 2.1 ± 2.4 mV vs. sham EA, 0.3 ± 1.4 mV, *P* = 0.010, *Z* = −2.573; FDI: EA, 3.1 ± 3.3 mV vs. sham EA, 0.3 ± 2.4 mV, *P* = 0.012, *Z* = −2.513; Mann–Whitney *U*-tests). However, a significant reduction was observed after EA in the representation of the face (OO: pre-EA, 6.4 ± 4.5 mV vs. post-EA, 5.5 ± 4.0 mV, *P* = 0.034, *Z* = −2.119). No significant changes were observed in the upper arm and leg representations (DM: pre-EA, 8.4 ± 3.7 mV vs. post-EA, 9.1 ± 4.8 mV, *P* = 0.330, *Z* = −0.975; TA: pre-EA, 4.2 ± 2.1 mV vs. post-EA, 4.4 ± 2.2 mV, *P* = 0.455, *Z* = −0.747; Wilcoxon signed-rank tests).

### 3.2. Effect of EA on COG

The COGs of the representations mapped in the present study included the medial-to-lateral leg, upper arm, forearm, hand, and face representations. The COGs of the forearm (EIP: pre-EA, *x* = 6.0 ± 0.7, *y* = 7.8 ± 2.3) and hand (FDI: pre-EA, *x* = 6.0 ± 0.8, *y* = 7.6 ± 2.3) representations were in close proximity. We observed no significant changes in the COG of any representation after the intervention (all muscles: *P* > 0.05, Wilcoxon signed-rank tests) (Table 1).

### 3.3. Effect of EA on Map Area

We observed no significant change in map area for any representation following the intervention (all muscles: *P* > 0.05, Wilcoxon signed-rank tests) (Table 1).

### 3.4. Within-Representation Plasticity

EA at TE5 on the forearm increased the map volume of the forearm muscle (EIP), indicating increased excitability in the forearm representation (Figure 3(a)). Early studies reported that changes in MEP amplitude induced by somatosensory stimulation were supraspinal [31–33] and were affected by *γ*-aminobutyric acid (GABA)-ergic mechanisms [34]. Thus, the increase in map volume induced by EA was not the result of facilitation in the spinal circuits, but rather facilitation in the motor cortex. EA at TE5 on the forearm resulted in considerable increases in the map volume of the cortical motor representation in the absence of changes in the area and COG of the motor map. This indicates that EA led to a change in cortical excitability rather than in the distribution of the representation. Changes in map area and COG may occur as a result of long-term somatosensory stimulation or long-term deprivation of somatosensory input after amputation. In healthy subjects, long-term daily TENS of muscle in the hand [6] or daily training of a coordinated movement [15] results in an enlargement in cortical representations of the target muscles. In amputees, the representations of proximal limb have been shown to expand into the deafferented cortex (representing the hand) and cortical stimulation of the hand representation has been demonstrated to evoke contraction of the amputated stump [35, 36]. Thus, the absence of changes in the area and COG may be due to the short-term stimulation applied in the present study.

### 3.5. Plasticity between Forearm and Hand Representations

Forearm stimulation increased the map volume of the forearm (EIP) representation and had a simultaneous analogous effect in the hand (FDI) representation (Figure 3(a)), suggesting EA-induced coactivation of the forearm and hand representations. The results obtained in this study are similar to those reported in previous studies. One study found that after the removal of the needle on LI11 and TE5 (both located on the forearm), the MEP amplitude of FDI was significantly increased compared to baseline [37]. Moreover, peripheral nerve stimulation at the wrist was shown to simultaneously induce marked changes in the cortical excitability of multiple hand muscles [5]. This plasticity may be closely related to the extensive horizontal intrinsic connectivity between these representations in the motor cortex [3, 38]. The internal organization of these representations within the motor cortex is best described as a network having a broadly distributed overlap. Intracortical microstimulation in one position within forelimb representation was shown to simultaneously induce movement in the digits and wrist [39, 40]. Moreover, the COGs of the hand and forearm representations were found to be in close proximity in present study (Table 1), indicating high levels of overlap between these two areas. Many studies have reported overlapping activation between the forearm and hand representations in the human motor cortex. For example, one fMRI study observed that these areas exhibited shared activation following hand and forearm movement [2]. Additionally, a TMS multiple-muscle mapping study reported a >70% overlap between hand and forearm muscles [41]. However, previous studies have shown a reciprocal inhibitory effect between the forearm and hand representations in the motor cortex. An extension and lateral shift of the stump muscle representation was reported in patients with long-standing upper limb amputation [42]. Additionally, an animal study reported an expanded hand representation and a contracted forearm representation in monkey motor cortex after long-term hand training, while the reverse was observed after long-term forearm training [43].

Taken together, the above studies indicate the existence of a specific activity pattern between forearm and hand representations, with both coactivation and reciprocal inhibition due to horizontal intrinsic connectivity.

### 3.6. Plasticity between Forearm and Face Representations

Stimulation of the forearm not only increased the map volume of the forearm (EIP) and hand (FDI) representations but also reduced the map volume of the face (OO) representation (Figure 3(b)). This indicates an inhibitory influence of EA on face representation, expressed as decreased motor cortical excitability. Both animal and human data on cortical plasticity indicate that changes in somatosensory input can induce plasticity [44, 45]. There is an inhibitory effect between the forearm and face representations of the motor cortex; for example, patients with traumatic forearm amputation show a medial shift of their face representation toward the forearm representation [46, 47]. Following facial nerve transection in the rat, forelimb movement can be evoked from some sites where only vibrissal movement could be elicited before nerve transection, suggesting an expanded region of forelimb representation within the previous face representation in the motor cortex [48]. The hand representation of patients with facial palsy expands toward the face representation in the motor cortex contralateral to the affected side [49]. Conversely, the reduced size of the hand representation contralateral to the affected side is reversed following the application of botulinum toxin in patients with hemifacial spasm [50].

In the present study, enhanced somatosensory input following EA effectively induced an inhibitory effect between the forearm and face representations under physiological conditions. Previous research has also reported an inhibitory effect between pharynx and oesophagus representations after somatosensory stimulation of the pharynx [51]; however, the mechanisms of this inhibitory effect are unclear. Latent intracortical connections have been demonstrated to be responsible for rapid reorganization between vibrissae and forelimb representations in the motor cortex following rat facial nerve transection [48].

### 3.7. Interactions between Forearm and Upper Arm Representations

In the present study, stimulation of the forearm did not significantly affect the map volume of the upper arm representation (Table 1), which differs from the EA-induced plasticity between the forearm and hand/face representations. In human somatotopography, the forearm and upper limb representations are adjacent to one another [52]. An overlap between the forearm and upper arm representations has also been demonstrated in fMRI studies of the human motor cortex [2]. Patients with phantom limb pain after upper arm amputation have increased cortical excitability and a larger territory of upper arm representation on the amputated side compared to those of the intact side, indicating reorganization between the upper arm and forearm representations [53]. Nevertheless, a TMS multiple-muscle mapping study observed a high overlap for forearm–hand muscles and a low overlap for upper arm–forearm muscles, which may explain why the upper arm representation was not facilitated by forearm stimulation in the present study [41]. Stimulation parameters, such as the intensity, frequency, and time course, have a crucial influence on the somatosensory stimulation-induced plasticity [54]. In contrast to our findings, a previous study reported no significant changes in motor cortical excitability after 90s of manual acupuncture in healthy subjects [55]. Thus, the EA stimulation parameters used in this study may not have been sufficient to induce plasticity between the forearm and upper arm representations.

### 3.8. Interactions between Forearm and Leg Representations

The results of this study show that EA stimulation of the forearm did not affect the map volume of the leg representation, suggesting a lack of somatosensory-induced plasticity between the forearm and leg representations. This may be due to the great anatomical distance between the forearm and the leg. Our results show that the COGs of the forearm and leg representations are poles apart. Moreover, the leg and forearm representations are separated by the arm and trunk representations, with no horizontal intrinsic connectivity between the representations of the leg and forearm.

## 4. Conclusions

The findings of the present study highlight the potential of somatosensory stimulation as a useful complementary therapy for neurological or facial diseases. We have shown that the (facilitatory or inhibitory) plasticity changes in cortical excitability induced by somatosensory stimulation were not restricted to the stimulated area but also extended to other areas. This may relate to the extensive horizontal intrinsic connectivity between different representations in the primary motor cortex.

## Figures and Tables

**Table 1:** Mean (±SD) map area and mean (±SD) COG pre- and postintervention for the EA and sham EA groups.

Measures	Muscle	EA		Sham EA	
		Pre	Post	Pre	Post
Area (cm^2^)	EIP	15.9 ± 4.2	16.2 ± 4.2	14.5 ± 2.5	13.3 ± 2.9
	FDI	13.8 ± 3.3	14.9 ± 4.7	13.5 ± 2.7	14 ± 3.3
	OO	22.2 ± 9.8	20.0 ± 8.1		
	DM	19.6 ± 4.7	18.6 ± 4.6		
	TA	14.6 ± 4.6	15.3 ± 4.5		
COG (*x*, *y*)	EIP	(6.0 ± 0.7, 7.8 ± 2.3)	(6.1 ± 0.8, 7.8 ± 2.3)	(4.7 ± 0.8, 7.4 ± 1.3)	(4.7 ± 0.7, 7.4 ± 1.2)
	FDI	(6.0 ± 0.8, 7.6 ± 2.3)	(6.2 ± 1.1,7.7 ± 2.3)	(5.3 ± 1.0, 7.7 ± 1.5)	(5.2 ± 0.9, 7.8 ± 1.3)
	OO	(7.2 ± 1.5, 6.2 ± 1.3)	(7.1 ± 1.7, 6.1 ± 1.3)		
	DM	(4.5 ± 1.0, 8.7 ± 1.4)	(4.6 ± 1.2, 8.7 ± 1.6)		
	TA	(1.2 ± 0.6, 8.7 ± 1.5)	(1.0 ± 0.6, 8.7 ± 1.5)		

Abbreviations: COG: centre of gravity; DM: deltoid muscle; EA: electroacupuncture; EIP: extensor indicis proprius; FDI: first dorsal interosseous; OO: orbicularis oculi; SD: standard deviation; TA: tibialis anterior.

## Data Availability

All data included in this study are available from the corresponding author upon request.
